# Supplementary material for: Hot spot prediction in protein-protein interactions by an ensemble system
Source: BMC Syst Biol. 2018 Dec 31;12(Suppl 9):132. doi: 10.1186/s12918-018-0665-8 (PMC6311905; doi:10.1186/s12918-018-0665-8)
Supplement: Supplementary file 2 — All relevant MATLAB code. A simple Matlab implement of our predictor. (ZIP 19,742 kb) [file 12918_2018_665_MOESM2_ESM.zip › Additional file 2/Read ASA/ASA website/dbMPIKT/4HMYC.html]

 NetSurfP - Protein Surface Accessibility and Secondary Structure Predictions

  

|  |  |  |
| --- | --- | --- |
|  |  | NetSurfP - Protein Surface Accessibility and Secondary Structure Predictions  Technical University of Denmark |

  


---

```
# For publication of results, please cite:
# A generic method for assignment of reliability scores applied to solvent accessibility predictions.
# Bent Petersen, Thomas Nordahl Petersen, Pernille Andersen, Morten Nielsen and Claus Lundegaard
# BMC Structural Biology 2009, 9:51 doi:10.1186/1472-6807-9-51
#
# Column 1: Class assignment - B for buried or E for Exposed - Threshold: 25% exposure, but not based on RSA
# Column 2: Amino acid
# Column 3: Sequence name
# Column 4: Amino acid number
# Column 5: Relative Surface Accessibility - RSA
# Column 6: Absolute Surface Accessibility
# Column 7: Z-fit score for RSA prediction
# Column 8: Probability for Alpha-Helix
# Column 9: Probability for Beta-strand
# Column 10: Probability for Coil
E M  4HMYC                  1    0.665 133.127  -2.445   0.003   0.003   0.994
E H  4HMYC                  2    0.621 112.978   0.340   0.005   0.015   0.979
E H  4HMYC                  3    0.600 109.049  -0.315   0.018   0.047   0.935
E H  4HMYC                  4    0.556 101.064  -0.933   0.018   0.047   0.935
E H  4HMYC                  5    0.471  85.693  -0.588   0.018   0.047   0.935
E H  4HMYC                  6    0.564 102.555   0.485   0.018   0.088   0.893
E H  4HMYC                  7    0.279  50.750   1.111   0.005   0.336   0.660
E E  4HMYC                  8    0.314  54.926   1.545   0.003   0.718   0.279
B M  4HMYC                  9    0.036   7.264   0.972   0.002   0.816   0.182
B R  4HMYC                 10    0.150  34.373   1.783   0.001   0.900   0.099
B I  4HMYC                 11    0.019   3.496   1.547   0.001   0.959   0.040
B L  4HMYC                 12    0.021   3.882   1.499   0.000   0.983   0.017
B M  4HMYC                 13    0.016   3.262   1.630   0.001   0.959   0.040
B V  4HMYC                 14    0.029   4.473   0.894   0.001   0.900   0.099
B G  4HMYC                 15    0.034   2.684  -0.676   0.004   0.514   0.481
B L  4HMYC                 16    0.134  24.517   0.098   0.004   0.085   0.910
E D  4HMYC                 17    0.365  52.597  -0.699   0.018   0.019   0.964
E A  4HMYC                 18    0.258  28.432  -1.293   0.058   0.017   0.925
B A  4HMYC                 19    0.047   5.146  -0.258   0.246   0.004   0.750
B G  4HMYC                 20    0.047   3.675  -0.992   0.430   0.016   0.555
B K  4HMYC                 21    0.091  18.801   0.038   0.802   0.014   0.185
B T  4HMYC                 22    0.100  13.856  -0.278   0.879   0.010   0.111
B T  4HMYC                 23    0.076  10.555   0.388   0.938   0.007   0.055
B I  4HMYC                 24    0.037   6.900   0.447   0.938   0.007   0.055
B L  4HMYC                 25    0.028   5.090   0.303   0.938   0.007   0.055
B Y  4HMYC                 26    0.235  50.113   1.058   0.950   0.028   0.022
B K  4HMYC                 27    0.141  28.963   1.254   0.911   0.033   0.057
B L  4HMYC                 28    0.071  13.018   0.398   0.831   0.044   0.125
B K  4HMYC                 29    0.194  39.803   0.646   0.660   0.049   0.291
E L  4HMYC                 30    0.595 108.963   0.306   0.257   0.016   0.727
E G  4HMYC                 31    0.588  46.244  -0.377   0.018   0.019   0.964
E E  4HMYC                 32    0.535  93.499   0.936   0.018   0.088   0.893
B I  4HMYC                 33    0.089  16.465  -0.153   0.020   0.205   0.775
E V  4HMYC                 34    0.449  69.057   0.211   0.020   0.205   0.775
E T  4HMYC                 35    0.636  88.144  -0.067   0.021   0.279   0.699
B T  4HMYC                 36    0.283  39.294  -0.092   0.022   0.359   0.619
E I  4HMYC                 37    0.308  57.054   0.746   0.004   0.420   0.576
B P  4HMYC                 38    0.170  24.151  -0.551   0.005   0.336   0.660
B T  4HMYC                 39    0.135  18.724  -1.153   0.004   0.616   0.381
B I  4HMYC                 40    0.097  17.871   0.321   0.003   0.718   0.279
B G  4HMYC                 41    0.158  12.466  -1.574   0.003   0.718   0.279
B F  4HMYC                 42    0.069  13.828   0.364   0.003   0.718   0.279
B N  4HMYC                 43    0.280  41.051   0.198   0.001   0.900   0.099
B V  4HMYC                 44    0.174  26.682   0.670   0.001   0.959   0.040
E E  4HMYC                 45    0.313  54.629   1.258   0.001   0.959   0.040
E T  4HMYC                 46    0.387  53.719   1.268   0.001   0.959   0.040
B V  4HMYC                 47    0.081  12.496   0.966   0.001   0.959   0.040
E E  4HMYC                 48    0.582 101.606   1.065   0.002   0.816   0.182
E Y  4HMYC                 49    0.444  94.968   0.025   0.005   0.262   0.733
E K  4HMYC                 50    0.701 144.216   0.462   0.004   0.085   0.910
E N  4HMYC                 51    0.538  78.719   1.243   0.005   0.262   0.733
B I  4HMYC                 52    0.054  10.045   0.967   0.001   0.900   0.099
E S  4HMYC                 53    0.294  34.480   2.515   0.001   0.959   0.040
B F  4HMYC                 54    0.027   5.439   1.021   0.006   0.962   0.032
B T  4HMYC                 55    0.078  10.777   1.473   0.006   0.962   0.032
B V  4HMYC                 56    0.024   3.689   0.976   0.006   0.962   0.032
B W  4HMYC                 57    0.063  15.103   0.407   0.001   0.900   0.099
B D  4HMYC                 58    0.058   8.358  -0.560   0.021   0.756   0.223
B V  4HMYC                 59    0.077  11.789  -0.021   0.064   0.216   0.721
B G  4HMYC                 60    0.129  10.137  -0.834   0.115   0.016   0.868
B G  4HMYC                 61    0.228  17.936  -1.314   0.257   0.016   0.727
B Q  4HMYC                 62    0.146  26.004   0.123   0.522   0.016   0.462
E D  4HMYC                 63    0.449  64.686  -0.162   0.622   0.015   0.363
E K  4HMYC                 64    0.310  63.829   0.324   0.622   0.015   0.363
B I  4HMYC                 65    0.056  10.286   0.382   0.622   0.015   0.363
B R  4HMYC                 66    0.225  51.479   0.657   0.751   0.050   0.199
E P  4HMYC                 67    0.288  40.881  -0.523   0.717   0.014   0.269
B L  4HMYC                 68    0.081  14.849  -0.045   0.717   0.014   0.269
B W  4HMYC                 69    0.056  13.540   0.767   0.717   0.014   0.269
E R  4HMYC                 70    0.408  93.432   0.873   0.802   0.014   0.185
B H  4HMYC                 71    0.261  47.440  -0.232   0.717   0.014   0.269
B Y  4HMYC                 72    0.084  18.058   0.359   0.717   0.014   0.269
B F  4HMYC                 73    0.073  14.731   0.472   0.561   0.047   0.393
E Q  4HMYC                 74    0.459  82.031   0.899   0.268   0.043   0.689
E N  4HMYC                 75    0.421  61.620  -0.588   0.053   0.043   0.903
B T  4HMYC                 76    0.026   3.648   0.633   0.018   0.088   0.893
B Q  4HMYC                 77    0.181  32.309   0.346   0.005   0.336   0.660
B G  4HMYC                 78    0.025   1.944   0.583   0.001   0.900   0.099
B L  4HMYC                 79    0.018   3.369   1.480   0.001   0.959   0.040
B I  4HMYC                 80    0.017   3.219   1.637   0.000   0.983   0.017
B F  4HMYC                 81    0.030   6.041   1.270   0.000   0.983   0.017
B V  4HMYC                 82    0.019   2.874   1.580   0.000   0.983   0.017
B V  4HMYC                 83    0.046   7.024   1.399   0.001   0.959   0.040
B D  4HMYC                 84    0.066   9.453   0.058   0.001   0.900   0.099
B S  4HMYC                 85    0.053   6.212   0.534   0.005   0.336   0.660
B N  4HMYC                 86    0.239  35.063   0.412   0.005   0.045   0.951
E D  4HMYC                 87    0.345  49.743   1.055   0.018   0.019   0.964
E R  4HMYC                 88    0.434  99.478   1.574   0.600   0.003   0.397
E E  4HMYC                 89    0.620 108.279   1.213   0.502   0.002   0.495
B R  4HMYC                 90    0.114  26.198   0.603   0.502   0.002   0.495
B V  4HMYC                 91    0.046   7.086  -0.131   0.923   0.002   0.076
E N  4HMYC                 92    0.564  82.614   1.616   0.970   0.001   0.030
E E  4HMYC                 93    0.408  71.243   1.650   0.970   0.001   0.030
B A  4HMYC                 94    0.012   1.300   1.681   0.970   0.001   0.030
E R  4HMYC                 95    0.343  78.593   1.349   0.970   0.001   0.030
E E  4HMYC                 96    0.540  94.373   2.072   0.970   0.001   0.030
B E  4HMYC                 97    0.080  14.046   0.837   0.988   0.000   0.012
B L  4HMYC                 98    0.040   7.342   0.541   0.988   0.000   0.012
E M  4HMYC                 99    0.438  87.664   1.747   0.988   0.000   0.012
E R  4HMYC                100    0.462 105.798   1.829   0.970   0.001   0.030
B M  4HMYC                101    0.079  15.708   0.354   0.970   0.001   0.030
B L  4HMYC                102    0.106  19.354   0.032   0.923   0.002   0.076
E A  4HMYC                103    0.632  69.679   1.288   0.858   0.002   0.139
E E  4HMYC                104    0.574 100.225   0.480   0.782   0.003   0.216
E D  4HMYC                105    0.437  62.986  -0.672   0.502   0.002   0.495
E E  4HMYC                106    0.444  77.514  -0.565   0.176   0.004   0.820
E L  4HMYC                107    0.342  62.639  -1.065   0.053   0.005   0.942
E R  4HMYC                108    0.696 159.338   0.188   0.016   0.005   0.979
E D  4HMYC                109    0.630  90.826   0.677   0.005   0.015   0.979
B A  4HMYC                110    0.027   3.030   1.283   0.004   0.138   0.858
B V  4HMYC                111    0.073  11.205   0.644   0.002   0.816   0.182
B L  4HMYC                112    0.030   5.511   1.029   0.001   0.959   0.040
B L  4HMYC                113    0.015   2.728   1.814   0.000   0.983   0.017
B V  4HMYC                114    0.012   1.798   1.847   0.001   0.959   0.040
B F  4HMYC                115    0.016   3.131   1.486   0.001   0.959   0.040
B A  4HMYC                116    0.016   1.785   0.761   0.003   0.718   0.279
B N  4HMYC                117    0.041   6.076  -0.122   0.004   0.420   0.576
B K  4HMYC                118    0.100  20.632   0.504   0.019   0.141   0.840
B Q  4HMYC                119    0.133  23.682   0.226   0.018   0.088   0.893
B D  4HMYC                120    0.125  17.955  -0.936   0.018   0.047   0.935
B L  4HMYC                121    0.117  21.459   0.441   0.115   0.016   0.868
E P  4HMYC                122    0.526  74.611   0.639   0.109   0.005   0.886
E N  4HMYC                123    0.707 103.490  -0.547   0.053   0.005   0.942
E A  4HMYC                124    0.440  48.488  -0.716   0.109   0.005   0.886
B M  4HMYC                125    0.320  63.992  -0.449   0.109   0.005   0.886
E N  4HMYC                126    0.463  67.812   0.148   0.058   0.017   0.925
E A  4HMYC                127    0.397  43.705   0.090   0.858   0.002   0.139
E A  4HMYC                128    0.579  63.795   0.522   0.879   0.010   0.111
E E  4HMYC                129    0.434  75.890   1.561   0.879   0.010   0.111
B I  4HMYC                130    0.036   6.641   0.338   0.802   0.014   0.185
E T  4HMYC                131    0.264  36.672   1.682   0.622   0.015   0.363
E D  4HMYC                132    0.543  78.232   1.145   0.970   0.001   0.030
E K  4HMYC                133    0.512 105.257   0.702   0.970   0.001   0.030
E L  4HMYC                134    0.282  51.598   0.634   0.970   0.001   0.030
B G  4HMYC                135    0.166  13.064   0.184   0.923   0.002   0.076
E L  4HMYC                136    0.305  55.882   1.298   0.923   0.002   0.076
E H  4HMYC                137    0.585 106.430   1.401   0.923   0.002   0.076
B S  4HMYC                138    0.154  18.014   0.845   0.923   0.002   0.076
B L  4HMYC                139    0.033   5.987  -1.354   0.858   0.002   0.139
E R  4HMYC                140    0.684 156.682   1.368   0.782   0.003   0.216
E H  4HMYC                141    0.771 140.281   0.738   0.502   0.002   0.495
E R  4HMYC                142    0.294  67.280   1.269   0.058   0.017   0.925
E N  4HMYC                143    0.614  89.846   0.979   0.005   0.015   0.979
B W  4HMYC                144    0.043  10.390   0.636   0.004   0.197   0.799
E Y  4HMYC                145    0.212  45.304   1.674   0.001   0.900   0.099
B I  4HMYC                146    0.041   7.677   0.554   0.001   0.959   0.040
B Q  4HMYC                147    0.066  11.841   0.561   0.001   0.959   0.040
B A  4HMYC                148    0.073   8.023  -0.510   0.001   0.959   0.040
B T  4HMYC                149    0.023   3.218   0.707   0.002   0.816   0.182
B C  4HMYC                150    0.055   7.694  -0.195   0.004   0.616   0.381
B A  4HMYC                151    0.032   3.515  -1.370   0.022   0.359   0.619
B T  4HMYC                152    0.233  32.386   0.424   0.019   0.141   0.840
E S  4HMYC                153    0.507  59.467   0.695   0.018   0.047   0.935
E G  4HMYC                154    0.450  35.415  -1.246   0.115   0.016   0.868
E D  4HMYC                155    0.487  70.162   0.939   0.257   0.016   0.727
B G  4HMYC                156    0.184  14.465   0.469   0.181   0.016   0.803
B L  4HMYC                157    0.024   4.321   1.329   0.923   0.002   0.076
E Y  4HMYC                158    0.354  75.543   1.701   0.970   0.001   0.030
E E  4HMYC                159    0.370  64.726   1.900   0.970   0.001   0.030
B G  4HMYC                160    0.026   2.007   1.012   0.988   0.000   0.012
B L  4HMYC                161    0.021   3.808   1.337   0.988   0.000   0.012
E D  4HMYC                162    0.340  49.008   2.377   0.988   0.000   0.012
E W  4HMYC                163    0.276  66.474   2.089   0.988   0.000   0.012
B L  4HMYC                164    0.023   4.211   1.190   0.988   0.000   0.012
B S  4HMYC                165    0.038   4.442   0.361   0.988   0.000   0.012
E N  4HMYC                166    0.534  78.163   2.077   0.988   0.000   0.012
E Q  4HMYC                167    0.319  57.027   2.171   0.970   0.001   0.030
B L  4HMYC                168    0.022   4.101   0.792   0.970   0.001   0.030
E R  4HMYC                169    0.343  78.433   1.419   0.923   0.002   0.076
E N  4HMYC                170    0.769 112.508   1.822   0.782   0.003   0.216
E Q  4HMYC                171    0.551  98.426   1.296   0.502   0.002   0.495
E K  4HMYC                172    0.727 149.544   0.309   0.003   0.003   0.994

Explain the output.  Go back.
```

---
